# P2X7 Receptor in Hematological Malignancies

**DOI:** 10.3389/fcell.2021.645605

**Published:** 2021-03-05

**Authors:** Elena De Marchi, Anna Pegoraro, Elena Adinolfi

**Affiliations:** Department of Medical Sciences, University of Ferrara, Ferrara, Italy

**Keywords:** P2X7 receptor, ATP, leukemia, lymphoma, multiple myeloma

## Abstract

The P2X7 receptor is an ion channel gated by the nucleotide ATP, known for its role in immune responses and recently emerging as a critical onco-promoting factor. Lymphocytes, myeloid cells, and their precursors were among the first cells proved to express a functional P2X7 receptor; therefore, it is not surprising that lymphoproliferative and myeloproliferative diseases, also known as hematological malignancies, were shown to be related in their insurgence and progression to P2X7 alterations. Here, we overview established and recent literature relating P2X7 with the biological mechanisms underlying leukemias, lymphomas, and multiple myeloma development. Particular attention is paid to studies published in the very recent past correlating P2X7 with ATP concentration in the leukemic microenvironment and P2X7 overexpression to acute myeloid leukemia aggressiveness and response to chemotherapy. The described literature strongly suggests that P2X7 and its genetic variants could be regarded as potential new biomarkers in hematological malignancies and that both P2X7 antagonists and agonists could emerge as new therapeutic tools alone or in combination with traditional chemotherapy.

## Introduction

Extracellular ATP represents a pivotal and abundant biochemical component of the tumor microenvironment, affecting cancer cell proliferation, and interaction with the immune system, as well as being the primary source of adenosine. The balance between extracellular ATP and adenosine is central in determining cancer progression, as, while ATP promotes antitumoral immune response, adenosine is a known immunosuppressive mediator, facilitating tumor immune escape (Di Virgilio and Adinolfi, 2017; Vijayan et al., 2017; Di Virgilio et al., 2018a). The involvement in carcinogenesis of CD39 and CD73 ectonucleotidases, responsible for ATP degradation and the consequent adenosine generation, is well recognized (Feng et al., 2011; Stagg et al., 2012). Accordingly, inhibitors of ATP hydrolyzing enzymes CD39, CD73, and adenosine A2A receptor antagonists, which are considered the next generation of immune checkpoint inhibitors, significantly reduce tumor growth by improving T cell-mediated responses (Vijayan et al., 2017).

In recent years, a large amount of literature confirmed the role of ATP-gated P2X channels in tumor development and progression. In particular, the P2X7 receptor assumes multiple functions in cancer, as it favors tumor growth and dissemination and mediates vesicular release of pro-inflammatory factors from immune cells (Adinolfi et al., 2012b; Di Virgilio et al., 2017). P2X7 is an ATP-gated ion channel triggering Na^+^ and Ca^2+^ influx and K^+^ efflux through the plasma membrane. The receptor’s subunits have two transmembrane domains divided by a large extracellular loop and intracellular N and C termini (North, 2002). P2X7, upon short ATP stimulation (2s or less), leads to a small cation channel opening. Moreover, when its activation is prolonged (4s or above), and the receptor is exposed to high ATP levels, the P2X7 opens an unselective membrane pore permeable to large hydrophilic molecules (Surprenant et al., 1996; Lara et al., 2020). The long intracellular carboxyl tail of P2X7 allows for macropore formation, as its deletion causes a loss of cytotoxic activity, not affecting the small ion channel (Surprenant et al., 1996; Di Virgilio et al., 2018b). Different P2X7 splice variants have been identified in humans, including just two functional ion channels: the full-length P2X7A isoform and the P2X7B isoform missing the C terminal domain (Cheewatrakoolpong et al., 2005; Adinolfi et al., 2010). Both P2X7A and B exert a growth-promoting activity (Adinolfi et al., 2010; Giuliani et al., 2014). As a result of its activation level, P2X7 can support cell proliferation or can be implicated in cell death (Di Virgilio et al., 1989, 1998; Baricordi et al., 1996; Surprenant et al., 1996; Di Virgilio and Adinolfi, 2017). Interestingly, P2X7 in its macropore conformation is a conduit for ATP efflux from the cell as confirmed by *in vivo* experiments proving that the concentration of extracellular ATP, released during allograft rejection, is higher in P2X7 wild-type mice than in their null counterpart (Pellegatti et al., 2005; Adinolfi et al., 2010, 2015a; Amores-Iniesta et al., 2017). Macropore opening can enhance the intracellular uptake of drugs, such as chemotherapeutic agents (Pacheco et al., 2016; Alves et al., 2018; Pegoraro et al., 2020).

It is now well acknowledged that P2X7 is expressed on hematopoietic and immune cells such as macrophages, dendritic cells, B and T lymphocytes, and thymocytes (Di Virgilio et al., 2017). Moreover, expression and activity of P2X7 are increased in numerous forms of solid cancers (Di Virgilio et al., 2009; Adinolfi et al., 2012b, 2015b; Amoroso et al., 2015; Gilbert et al., 2018). In line with these data, the first correlation between P2X7 and oncogenesis was revealed 30 years ago in a hematopoietic lymphoproliferative disorder, B chronic lymphocytic leukemia (B-CLL) (Wiley and Dubyak, 1989). Several successive research pieces confirmed P2X7 involvement in onco-hematological conditions, such as different forms of leukemia, lymphoma, and myeloma. Moreover, numerous roles of P2X7 in hematopoietic cells, including infection, inflammation, and the previously mentioned cell death or survival, have already been widely described, enough to make P2X7 a promising pharmacological target (Filippin et al., 2020).

In this review, we wish to give an overview of the prominent and current literature referring to the participation of P2X7 in hematological malignancies.

## P2X7 Role in Leukemias

Leukemia is a cancer of the early blood-forming cells, and either the bone marrow or the lymphatic system is involved. Some forms of leukemia are more common in children; other forms occur mostly in adults. Leukemia can be subdivided into acute (fast-growing) or chronic (slow-growing) and can also start from myeloid or lymphoid cells (Arber et al., 2016). Therefore, four main leukemia groups are recognized: acute lymphoblastic leukemia, acute myeloid leukemia (AML), chronic lymphocytic leukemia (CLL), and chronic myeloid leukemia.

**FIGURE 1:**
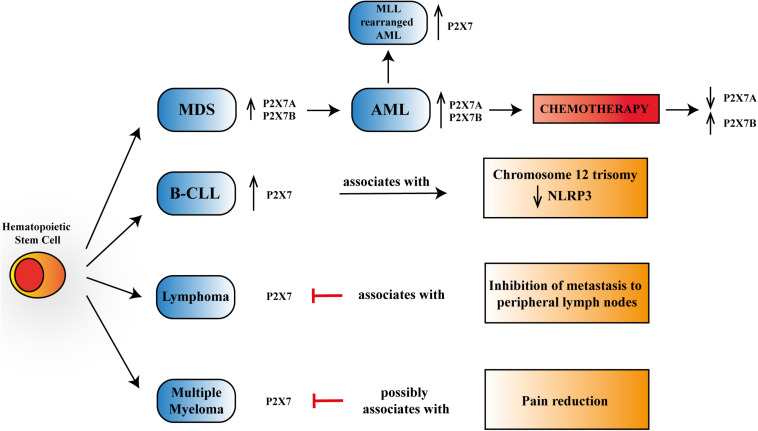
Schematic representation of P2X7 reported activities in myelodysplastic syndrome (MDS), acute myeloid leukemia (AML), B chronic lymphocytic leukemia (B-CLL), lymphoma, and multiple myeloma. P2X7 is upregulated, progressing from progenitor stem cells to MDS and overt AML, further increasing its expression in mixed-lineage leukemia (MLL)-rearranged AML. Chemotherapy leads to a reduction of P2X7 isoform A, which *via* macropore formation facilitates daunorubicin cytotoxicity while upregulating P2X7 isoform B that protects leukemic blasts from cell death. P2X7 expression rises in aggressive B-CLL and associates with chromosome 12 trisomy and a reduction in NLRP3 (NOD-, LRR,- and pyrin domain-containing protein 3) expression. In lymphoma, P2X7 blockade reduces metastasis to peripheral lymph nodes. In multiple myeloma, P2X7 antagonism could lead to pain reduction.

As mentioned above, B-CLL was the first model in which a correlation between P2X7 and oncogenesis was reported dating back to Wiley and Dubyak (1989). Some years later, a different work showed that P2X7 was upregulated in lymphocytes from patients with the aggressive variants of B-CLL and that incubation of these lymphocytes in the presence of ATP prevented their proliferation (Adinolfi et al., 2002). These data were recently confirmed by analyzing an extensive database of hematological malignancies showing higher expression of P2X7 in CLL than in healthy bone marrow controls (Feng et al., 2020; Figure 1). Many studies trying to associate the P2X7 loss-of-function polymorphism 1513A > C and B-CLL aggressiveness or progression were published in the same years, not always reaching similar conclusions. Indeed, while the polymorphism was associated with increased capacity to escape apoptosis and, therefore, to B-CLL bad prognosis (Wiley et al., 2002), it was also claimed to be protective in the advanced phases of the disease (Thunberg et al., 2002). These two studies (Thunberg et al., 2002; Wiley et al., 2002) lead to an extensive analysis of 1513A > C P2X7 variant in larger B-CLL cohorts, which failed to prove any positive or negative association of this polymorphism with disease progression (Thunberg et al., 2002; Starczynski et al., 2003; Zhang et al., 2003). These discording data, which could be due to limited knowledge at that time of other polymorphisms of the receptor or the presence of P2X7 splice variants, caused a long stop in the research trying to relate the receptor and B-CLL until a recent study that investigated the involvement of P2X7/NLRP3 (NOD-, LRR-, and pyrin domain-containing protein 3) inflammasome axis in this hematological condition (Salaro et al., 2016). P2X7-dependent NLRP3 inflammasome activation causing pro-inflammatory cytokine [interleukin (IL)-1β and IL-18] maturation and release is an accepted assumption (Orioli et al., 2017). However, the study by Salaro et al. (2016) demonstrated that in leukemic cells, NLRP3 is negatively regulating P2X7 and exerts an antiproliferative action, as its silencing promoted overexpression of the receptor and cell growth. Accordingly, P2X7 was overexpressed in B-CLL samples and associated with trisomy of chromosome 12, while NLRP3 seemed to be a protective factor for the disease (Salaro et al., 2016; Figure 1).

Overexpression and increased function of P2X7 were also reported in acute lymphoblastic and chronic myeloid leukemia patients (Zhang et al., 2004; Chong et al., 2010). However, the highest number of studies present in literature is related to P2X7’s role in AML of infancy and adulthood and to myelodysplastic syndromes (MDSs) (Zhang et al., 2004; Chong et al., 2010; Salvestrini et al., 2012, 2017; Lecciso et al., 2017; Feng et al., 2020; He et al., 2020; Pegoraro et al., 2020). AML is a severe hematological disease characterized by clonal expansion of hematopoietic stem cells, which, in some cases, can arise from a preexisting MDS (Richard-Carpentier and DiNardo, 2019). Both AML and MDS have been shown to express a functional P2X7 receptor and its best-characterized splice variants: P2X7A and P2X7B (Pegoraro et al., 2020; Figure 1). Interestingly, the levels of both P2X7 isoforms were upregulated in AML compared to MDS (Pegoraro et al., 2020), thus suggesting a role for P2X7 in malignant transformation (Pegoraro et al., 2020). These data are supported by the analysis of different genetic databases and patients’ specimens demonstrating an upregulation of P2X7 in AML cohorts (Chong et al., 2010; Salvestrini et al., 2017; Feng et al., 2020; He et al., 2020). Consistently, high P2X7 levels are associated with reduced overall survival (He et al., 2020). Moreover, Feng et al. (2020) revealed an increased expression of P2X7 in AML patients carrying chromosomal translocations of mixed-lineage leukemia (MLL) genes (MLL-rearranged AML), which are generally presented with unfavorable clinical outcomes (Figure 1).

Consistently, administration of different P2X7 antagonists caused a significant reduction in AML growth and dissemination (De Marchi et al., 2019; He et al., 2020; Pegoraro et al., 2020), as well as an increase in overall survival (He et al., 2020) in various murine models of the disease, obtained both in xenotransplanted and syngeneic mice, thus confirming the efficacy of P2X7 blockade both on human leukemic cells and in hosts with a functional immune system. Similar results were obtained when silencing the P2X7 receptor in both leukemic cell lines and leukemia-initiating stem cells (Feng et al., 2020; He et al., 2020), thus reproducing the effect of P2X7 ablation in solid cancer models (Adinolfi et al., 2012b). On the contrary, P2X7 overexpression increased the proliferative potential of leukemic blasts and the number of leukemia-initiating lymphocytes central for AML relapse (Feng et al., 2020; He et al., 2020). Various signaling proteins and circulating cytokines, activated by the P2X7 receptor, associate with increased tumorigenic activity in AML. These include c-myc (Pegoraro et al., 2020), Pre-B-cell leukemia transcription factor 3 (Pbx3) (Feng et al., 2020), cAMP Response Element-Binding Protein (CREB), D-3-phosphoglycerate dehydrogenase (PHGDH) (He et al., 2020), IL-1β, tumor necrosis factor alpha (TNFα), and interferon gamma (IFNγ) (De Marchi et al., 2019).

**FIGURE 2:**
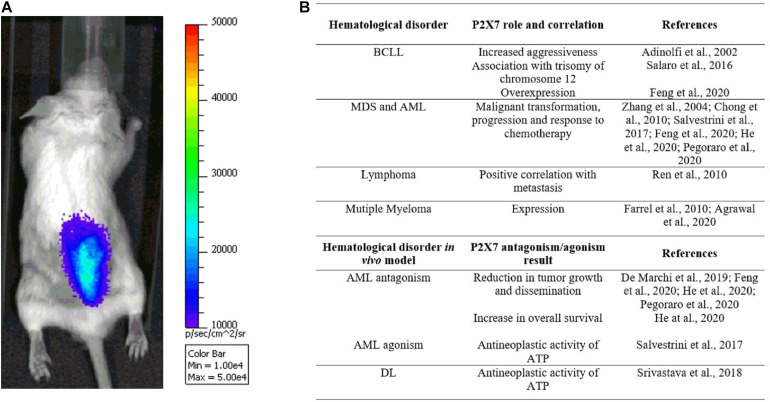
**(A)** The pmeLUC probe allows for live imaging of ATP in the leukemic microenvironment (for detailed methods, see De Marchi et al., 2020). Note the prevalent concentration of ATP and leukemic cells, injected 7 days before the acquisition in the right hind, at the spinal column, suggesting a tropism of acute myeloid leukemia (AML) cells to the backbone. **(B)** Table summarizing studies reporting P2X7 genetic association with hematological malignancies and efficacy of receptor’s blockade/activation in *in vivo* murine models.

Interestingly, the levels of extracellular ATP are also high in the AML microenvironment and tend to be concentrated in the spinal column area (Figure 2A; Lecciso et al., 2017; De Marchi et al., 2020; He et al., 2020), especially at the endosteal niche (He et al., 2020). Moreover, ATP in the AML milieu decreases upon P2X7 knockdown (De Marchi et al., 2019) while increasing following chemotherapy with daunorubicin (Lecciso et al., 2017; Pegoraro et al., 2020). A role for P2X7 in regulating AML response to chemotherapeutics classically administered in AML, such as daunorubicin and cytarabine, was also suggested by different groups (Lecciso et al., 2017; Salvestrini et al., 2017; Feng et al., 2020; Pegoraro et al., 2020). Indeed, as a member of the anthracycline family, daunorubicin is known to cause the activation of immunogenic cell death, a process involving the release from dying tumor cells of molecules acting as damage-associated molecular patterns (DAMPs), which includes ATP (Kepp et al., 2017; Lecciso et al., 2017). This is also the case of AML, where only daunorubicin and not cytarabine causes an increase of ATP and the main cytokines associated with immunogenic cell death: IFNγ, IL-1β, IL-2, and IL-12 (Lecciso et al., 2017). Interestingly, at the same time, ATP, released upon daunorubicin treatment, also promotes the immunosuppressive action of CD4^+^, CD25^+^, Foxp3^+^ regulatory T (Treg) cells but only in P2X7-expressing mice (Lecciso et al., 2017). Indeed, P2X7 is central in regulating Treg cells and their fitness markers in the tumor microenvironment, acting at the ATP levels *via* degrading enzyme CD73 (Adinolfi et al., 2019; De Marchi et al., 2019).

Finally, P2X7 also plays a role in the permeabilization of AML blasts to daunorubicin, facilitating the entry of the chemotherapeutic and, therefore, its toxicity (Pegoraro et al., 2020). This permeabilizing effect is specifically played by P2X7 isoform A, that it is consequently downregulated following chemotherapy in both AML relapsing and remitting patients. Simultaneously, the daunorubicin-dependent ATP release facilitates the proliferation of AML blasts expressing the P2X7B isoform. Indeed, P2X7B cannot form the macropore but still behaves like a small ion channel, promoting cell proliferation and protection from chemotherapy toxicity and, therefore, favoring AML relapse (Pegoraro et al., 2020). Consequently, P2X7B is upregulated in AML relapsing subjects and emerged as a potential biomarker and therapeutic target for this patient subset (Pegoraro et al., 2020). The assumption that two different P2X7 splice variants could play contrasting roles in the presence of high ATP concentration can also help to reconcile the antitumoral effect played by both P2X7 antagonists and agonists (Salvestrini et al., 2017; De Marchi et al., 2019; Pegoraro et al., 2020) in reducing AML growth in murine models. It will also be interesting to analyze isoform expression in large cohort databases to understand whether they are differentially expressed in MLL-rearranged AML and, in general, in poor outcome patients. Finally, the role of ATP and P2X7 in shaping the bone marrow niche and mediating hematopoietic precursors or leukemic stem cell localization, mobilization, and their behavior following allogeneic transplantation surely deserves further understanding (Salvestrini et al., 2017; Adamiak et al., 2018; Koldej et al., 2018; Cuthbertson et al., 2020; He et al., 2020).

## P2X7 and Lymphoma

Lymphoma is a cancer of the lymphatic system involving lymph nodes, spleen, thymus gland, and bone marrow. Lymphoma can affect all those areas as well as other organs throughout the body. Two main subtypes of lymphoma are known: Hodgkin’s lymphoma (HL) and non-Hodgkin’s lymphoma (NHL). These two diseases differ in the lymphoid cells involved, as the first one is marked by the presence of Reed–Sternberg cells, which are not present in the second one (Brauninger et al., 2006). To our knowledge, there are not many studies correlating P2X7 and lymphoma growth and progression, although the expression of the receptor in T and B lymphoma cells is well known (Di Virgilio et al., 2017). A study dating back to 2010 demonstrated that the silencing and blockade of P2X7 cause inhibition of metastasis of lymphoid neoplasm cells to peripheral lymph nodes, suggesting that the receptor’s expression positively correlates with neoplasm metastasis (Ren et al., 2010; Figure 1). Consistently, the same group demonstrated that in lymphoma cell lines and patients’ specimens, P2X7 acts as an upstream regulator of T complex polypeptide 1 (TCP-1). This protein is a known folding regulator facilitating tumor metastasis (Jiang et al., 2015). Another recent exciting study has focused the attention on the correlation between the P2X7–NLRP3 inflammasome complex and Sjögren’s syndrome, in which there is a predisposition to develop NHL. Authors proposed the complex as a potential pathway involved in the degenerative process leading to NHL genesis *via* the increased production of IL-18 by P2X7 (Baldini et al., 2017). Additional research showing a relationship between P2X7 and lymphoma concerns an aggressive and metastatic murine form of this neoplasm called Dalton’s lymphoma (DL). The authors tested the antitumoral effect of silica particles carrying ATP plus doxorubicin and designed to target cancer in a pH-dependent fashion on DL growth and progression (Srivastava et al., 2018). Srivastava et al. (2018) demonstrated a good efficiency of the particles in reducing lymphoma burden and increasing overall survival of lymphoma-bearing mice, suggesting that this effect will be mediated by upregulation of P2X7 receptor and consequent cytotoxicity.

## P2X7 in Multiple Myeloma

Multiple myeloma (MM) is a cancer of plasma cells, which spreads in the bone marrow and crowds out healthy blood cells with the consequent reduction of platelets and red and white blood cells. While the tumor’s etiology is still unknown, a genetic component seems to be involved in MM that is the second most common blood cancer after leukemia. Moreover, it is also the most frequent cancer affecting the skeleton, and bone lesion development is usual (Roodman, 2010; Adinolfi et al., 2012a). In 90% of patients, MM complications lead to increased bone destruction and inadequate bone formation (Marino and Roodman, 2018). Myeloma cells in the bone marrow are responsible for osteolytic lesions without bone formation by the secretion of factors interacting with osteoclasts and osteoblasts (Roodman, 2011; Diaz-delCastillo et al., 2020). Despite several advances in treatment, nowadays, MM is still an incurable disease, and more than two-thirds of patients suffer from bone pain and skeletal-related events as fractures (Diaz-delCastillo et al., 2020). P2X7 antagonist administration is well known to reduce chronic neuropathic and inflammatory pain (Honore et al., 2006; Nelson et al., 2006; Jacobson et al., 2020). Moreover, there is a demonstrated correlation between P2X7 and cancer pain. Indeed, in women developing chronic pain after mastectomy, the gain-of-function 489C > T P2X7 polymorphism was associated with high-intensity pain and, consistently, the loss-of-function 835G > A variant with reduced suffering (Sorge et al., 2012). Therefore, the P2X7 receptor has a strong therapeutic potential to cure MM-associated bone pain (Adinolfi et al., 2012a; Figure 1). Few investigations have analyzed the possible association between P2X7 and MM growth and progression. This is surprising considering P2X7 involvement in B-lymphocyte signaling (Shemon et al., 2007; Pippel et al., 2015), immunoglobulin secretion (Sakowicz-Burkiewicz et al., 2013), as well as the modulation of osteoblastic and osteoclastic responses (Jorgensen et al., 2002; Grol et al., 2009). The expression of a functional and proapoptotic P2X7 in human MM cells was first reported 10 years ago (Farrell et al., 2010) and confirmed by recent research documenting P2X7 activity in six different myeloma cell lines (Agrawal et al., 2020). Genetic loss-of-function variants of P2X7 have also been linked with an augmented risk of MM (Vangsted et al., 2014). Nevertheless, a single-nucleotide polymorphism analysis of P2X7 in MM patients did not reveal any association with either osteolytic bone disease or vertebral fractures. Agrawal et al. (2020) recently suggested that P2X7 activated by high ATP concentrations can impair myeloma growth, inducing cell cycle arrest instead of apoptosis. Additionally, the authors hypothesized that P2X7 would affect the interaction among myeloma cells, osteoblasts, and osteoclasts, favoring mineralization and reversing osteoclastic resorption lines (Agrawal et al., 2020).

## Conclusion

Research-based evidence summarized in this review strongly suggests a crucial role for P2X7 in hematological malignancies (Figure 2B), thus opening the way to exploiting P2X7 blockade or activation as a possible therapeutic strategy in different blood cancers. An in-depth analysis of P2X7 genetic variants identifying them as potential biomarkers for hematological disorders will also be advised.

## Author Contributions

EM wrote and edited the original draft and prepared the figures. AP revised and edited the initial draft. EA wrote, edited, and gave final approval to the manuscript. All authors contributed to the article and approved the submitted version.

## Conflict of Interest

The authors declare that the research was conducted in the absence of any commercial or financial relationships that could be construed as a potential conflict of interest.
